# The role of attention in eye-movement awareness

**DOI:** 10.3758/s13414-018-1553-4

**Published:** 2018-07-02

**Authors:** Aoife Mahon, Alasdair D. F. Clarke, Amelia R. Hunt

**Affiliations:** 1 School of Psychology, University of Aberdeen, Aberdeen, UK; 2 Institute for Health Research, University of Bedfordshire, Luton, UK; 3 Department of Psychology, University of Essex, Colchester, UK

**Keywords:** Eye movements and visual attention, Visual awareness

## Abstract

People are unable to accurately report on their own eye movements most of the time. Can this be explained as a lack of attention to the objects we fixate? Here, we elicited eye-movement errors using the classic oculomotor capture paradigm, in which people tend to look at sudden onsets even when they are irrelevant. In the first experiment, participants were able to report their own errors on about a quarter of the trials on which they occurred. The aim of the second experiment was to assess what differentiates errors that are detected from those that are not. Specifically, we estimated the relative influence of two possible factors: how long the onset distractor was fixated (dwell time), and a measure of how much attention was allocated to the onset distractor. Longer dwell times were associated with awareness of the error, but the measure of attention was not. The effect of the distractor identity on target discrimination reaction time was similar whether or not the participant was aware they had fixated the distractor. The results suggest that both attentional and oculomotor capture can occur in the absence of awareness, and have important implications for our understanding of the relationship between attention, eye movements, and awareness.

The visual world is enriched with far more information than we can possibly process. To successfully interact with our environment, we must be able to select relevant information. Attention is what achieves this selection. In this study, we are specifically interested in spatial attention and its relationship to the execution of eye movements. Both eye movements and attention are involved in the process of selectively sampling information from the visual array for more detailed processing, to the exclusion of other information. The functional similarity between them has led to the intuitively appealing hypothesis that covert attention and eye movements exist on a continuum, with covert attention simply being a subthreshold eye movement (Klein, 1980; Rizzolatti, Riggio, Dascola, & Umiltá, 1987). Although a strong version of this hypothesis has not found wide support (e.g. Smith & Schenk, 2012), several studies have demonstrated that covert attention tends to be allocated to the location where an eye movement is about to be executed (e.g. Deubel & Schneider, 1996; Hoffman & Subramaniam, 1995; Kowler, Anderson, Dosher, & Blaser, 1995; Shepherd, Findlay, & Hockey, 1986). Based on these studies, it is widely believed that it is not possible to move the eyes without also moving attention.

If eye movements necessarily recruit attention, one might expect people’s awareness of their own eye movements to be reasonably high (although, as we will return to in the discussion, the nature of the relationship between attention and awareness is the subject of intense debate). But awareness of eye movements is extremely limited. Using a converging methods approach, we previously measured eye-movement awareness during three tasks: visual search of a complex illustration, naming objects in a photographic image, and moving the eyes to a simple, single target (Clarke, Mahon, Irvine, & Hunt, 2017). Based on the results of all three experiments, we reached the conclusion that people have many indirect strategies at their disposal to boost accuracy when asked to report on their own eye movements, but when these strategies are not available, the accuracy of reports is close to chance. When alternative strategies are available, people are moderately above chance in their ability to report on their own eye movements (Foulsham & Kingstone, 2013; Marti, Bayet, & Dehaene, 2015). Consistent with our conclusions, Võ, Aizenman, and Wolfe (2016) found that people were no better at remembering where they just looked in a scene relative to a baseline of where they think other people would be likely to look (see also Kok, Võ, Aizenman, & Wolfe, 2017), and people make systematic errors in reporting when an eye movement occurred (Hunt & Cavanagh, 2009).

Earlier research on eye-movement errors suggested people are unaware of these, even when they are quite large. Theeuwes, Kramer, Hahn, and Irwin (1998, 1999) investigated erroneous eye movements executed towards task-irrelevant sudden onsets. Participants were instructed to move their eyes to a single orange circle amongst red circle distractors. On half of the trials, an additional red circle appeared between the existing circles. Eye movements were directed to this sudden onset on 30% to 40% of trials, even though it was irrelevant to the task. The high prevalence of eye movements to the irrelevant onset, known as *oculomotor capture*, has been replicated many times (e.g. Belopolsky, Kramer, & Theeuwes, 2008; Born, Kerzel, & Theeuwes, 2011; Godijn & Theeuwes, 2002, 2003; Hunt, Olk, Mühlenen, & Kingstone, 2004; Hunt, Mühlenen, & Kingstone, 2007; Wu & Remington, 2003). At the end of their experiment, Theeuwes et al. (1998) informally asked participants if the sudden onset affected their eye-movement behaviour. Most participants reported being unaware of the abrupt onset, and no participants reported that their eye movements were affected or captured by it. Extending this further, Belopolsky et al. (2008) used a similar task, but after each trial, participants were asked if they looked directly at the target. People were able to report errors around two-thirds of the time. Although the results of these two studies are somewhat at odds (i.e. are participants unaware of *all* errors, or just some of them?) they do reinforce the conclusion that people have limited awareness of their own eye movements, even when they know that they will be asked to report on them, and even when these movements are large errors that negatively impact their performance.

Abrupt onsets and salient events tend to capture not only our eyes (e.g. Theeuwes et al., 1998, 1999) but also our attention (e.g. Jonides & Yantis, 1988; Theeuwes, 1994, 1995; Yantis & Jonides, 1984). The oculomotor capture paradigm grew from a previous paradigm developed by Theeuwes (1991, 1992, 1994), commonly referred to as the ‘irrelevant singleton’ (Yantis & Egeth, 1999) or ‘additional singleton’ paradigm (e.g. Simons, 2000). Similar to the oculomotor design, an irrelevant distractor singleton is shown with a relevant target singleton. To the extent that the distractor has captured the participant’s attention, they should produce slower reaction times towards the target singleton compared with no distractor trials, and this is indeed the case (Theeuwes, 1991, 1992, 1994; Theeuwes & Godijn, 2001). The conclusion that attention is reflexively drawn to the irrelevant singleton was reinforced by Theeuwes (1996) and Theeuwes, Atchley, and Kramer (2000), who manipulated the congruency of characters presented within the distractor and target singletons. Reaction times to identify the character in the target were slower (by about 20–30 ms) when the character inside the distractor was incongruent than when it was congruent, suggesting that spatial attention had been allocated to the distractor on at least some of the trials.

Coming back to oculomotor capture, why are participants aware of their eye-movement errors on some trials but not others? At least two factors may be important. First, awareness could simply be a function of the dwell time on the distractor: that is, the longer the participant fixates the distractor, the more likely they are to notice/report having fixated it. Increased dwell time on an erroneously fixated stimulus was related to increases in error awareness in studies by both Mokler and Fischer (1999) and Belopolsky et al. (2008). However, in these studies the distributions of dwell times overlap, with many unreported errors having longer dwell times than reported errors. Moreover, it is not possible to determine the causal direction of this effect: were participants aware they were making an error because they fixated the distractor for longer? Or did participants fixate the distractor for longer because they were aware they were making an error?

The second potentially important determinant of eye-movement error awareness could be attention. It has previously been asserted that attention *necessarily* precedes all eye movements. If this is the case, attention should precede erroneous eye movements to the same extent as goal-directed eye movements. Whether or not the participant reports awareness of the error on a particular trial should have no relationship to the extent to which attention was allocated to the distractor (as measured by congruency effects). However, some studies have suggested eye movements can be executed in the absence of attention (e.g. Stelmach, Campsall, & Herdman, 1997; Van der Stigchel & De Vries, 2015). An error might go undetected on trials where the eyes, but not attention, went to the onset. In this case, awareness of an error may be related to the extent to which attention was allocated to the distractor.

To try and answer the question of what determines awareness of an error, we used a version of the oculomotor capture paradigm (Theeuwes et al., 1998) that would produce a large number of eye-movement errors by increasing the target/distractor colour similarity (see Hunt, von Mühlenen, et al., 2007). In Experiment 1 we simply asked participants to report, after each trial, whether or not the eye movement they made on that trial was ‘good’, meaning it went directly from the central fixation to the target. The purpose of this experiment was to confirm that we would obtain eye-movement errors using our setup, and to estimate the extent to which people are aware of these errors. In the second experiment, we repeated Experiment 1, but now asked participants to make a speeded response to the orientation of a *C* presented inside the colour singleton target. We also presented an irrelevant *C* inside the onset distractor. We selected for analysis only those trials on which participants first fixated the sudden onset distractor, and then made a corrective saccade to the target. To the extent that attention is allocated to the onset distractor, the direction of the *C* inside the onset should influence responses (accuracy and reaction time) to the *C* inside the target. We can relate the size of this interference effect, as well as dwell time on the onset, to awareness of the error. If undetected errors are due to a lack of attention to the onset, we should see larger interference effects when errors are detected relative to when they are not.

## Experiment 1: Oculomotor capture

Participants in the original oculomotor capture experiments (Theeuwes et al., 1998) were reported to have been unaware of their eyes persistently being misdirected towards irrelevant sudden onsets. This conclusion was based on subjective reports collected from simply asking participants during debriefing if they were aware of their errors during the experiment. Later, Belopolsky et al. (2008) conducted an oculomotor capture experiment in which they asked participants if they looked directly to the target after each trial and found they were in fact able to report errors on around two-thirds of trials, contradicting the original conclusion. However, capture rates were quite low (~16%) compared with the original study (~40%), so it is possible that a rarer capture event is more noticeable. In Experiment 1 of the current study, we therefore sought to clarify the extent to which participants are aware of the accuracy of their own eye movements in an oculomotor capture experiment.

Given that participants are required to provide binary responses about whether their eye movement was ‘good’, a simple accuracy measure, such as percentage correct, is not suitable for characterising performance. This is because the number of correct eye movements executed will differ between participants. Each participant will also have their own particular response bias (e.g. to usually say the eye movement was good). We will therefore present our results using two statistics commonly used in the classification literature: precision and recall. If we are trying to classify ‘error’ saccades from ‘good’ saccades, then the definitions are as follows:

- Accuracy: the proportion of all items successfully classified.
- Precision: the proportion of trials classified as ‘not good’ that did in fact contain saccade errors.
- Recall: the proportion of trials with saccade errors that are accurately classified as ‘not good’.

We conducted the experiment with a group of naïve participants. We attempted to disguise the real purpose of the study by telling participants that we were asking them to report on their accuracy so that we could remove error trials from the data. However, to determine whether awareness of the purpose of the experiment mattered for performance and awareness, we also tested two of the authors in the same experiment to see if their results would differ from the naïve participants. To foreshadow, the authors’ data looked similar to naïve participants’ data both in terms of the proportion of trials on which oculomotor capture occurred, as well as the reported awareness of that capture.

### Method

#### Participants

Ten naïve participants (five females, *M*_age_ = 22.5 years, range: 19–27 years) took part in Experiment 1. Two authors of the current article also participated. All participants were members of the academic community at the University of Aberdeen. The experiment was conducted with the signed consent of each participant, and the protocol was approved by the Psychology Research Ethics Committee at the University of Aberdeen. All participants had normal or corrected-to-normal vision. Naïve participants were remunerated £5 for their time.

#### Stimuli and procedure

**Fig. 1:**
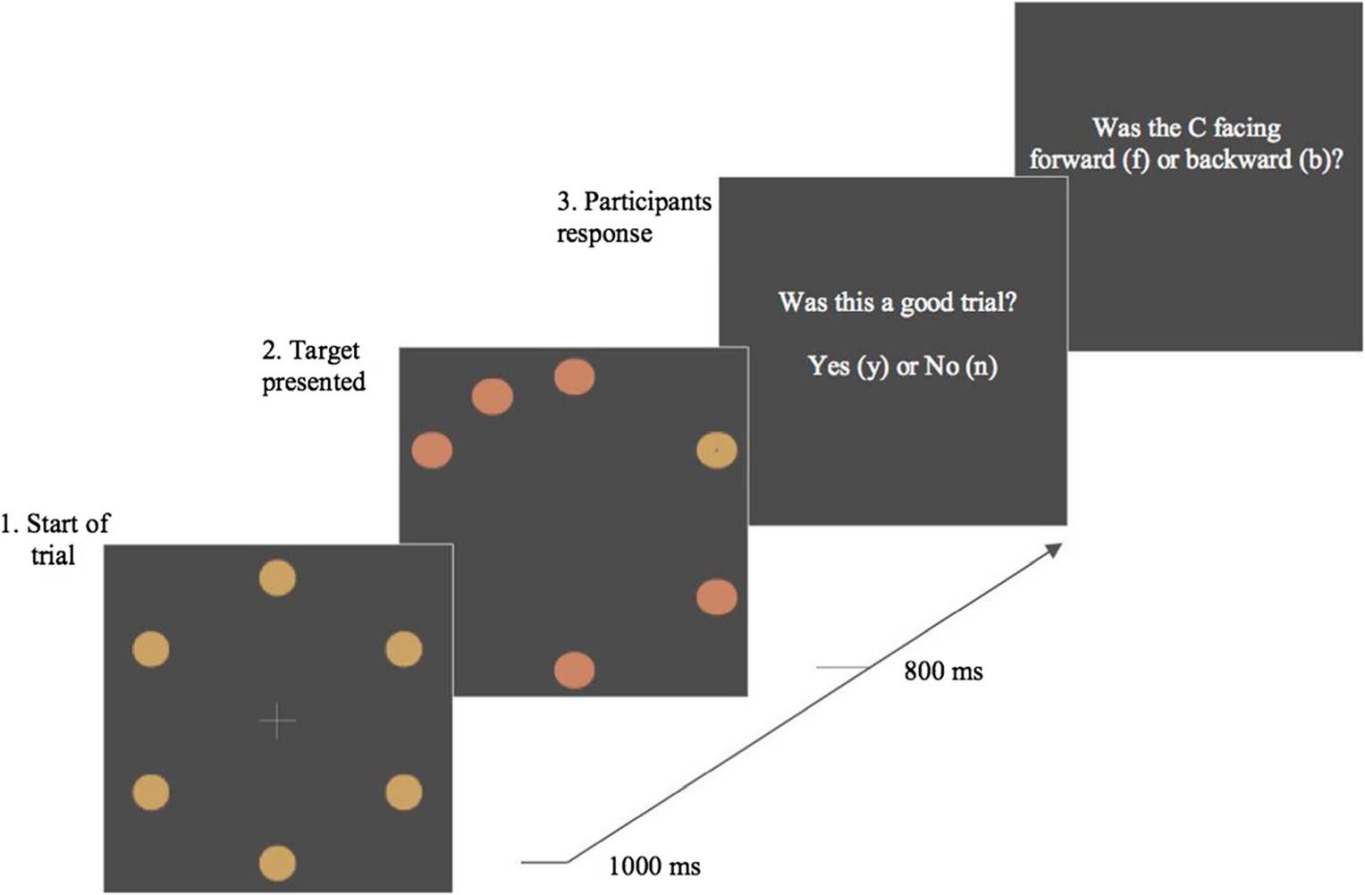
Example of an onset-present trial. Six orange circles are presented in a circular array around a central fixation cross (first panel). After 1,000 ms, five circles change to red, and one remains orange (second panel). Participants must saccade to the orange target and report the direction of the *C* inside. As this is an onset-present trial, an additional red circle appears at the same time as the colour change. After 800 ms, the trial terminates and participants are asked, ‘Was this a good trial?’. Responding ‘Yes’ means that the participant believed their eyes went directly to the orange circle on that trial; responding ‘No’ means that they believed their eyes did not go directly to the orange circle on that trial. If participants respond ‘Yes’ they are asked a second question: ‘Was the *C* facing forward (f) or backward (b)?’ (Colour figure online)

Experimental scripts were created and run using MATLAB with the PsychToolBox (Kleiner, Brainard, & Pelli 2007), and run on a PowerMac 10.8.2. All materials are publicly available on the Open Science framework (https://osf.io/an8gj/). Stimuli were presented on a Sony Trimaster EL OLED computer screen, 1920 × 1080 pixels. Participants’ heads were stabilized in a chin rest at a viewing distance of 57 cm. Participant responses were recorded using an Apple keyboard. Eye movements were monitored using an EyeLink 1000 (Ottawa, Canada) in the desktop configuration. The stimuli and procedure can be seen in Fig. 1.

Each trial began with a central fixation point on a blank grey screen (HSV coordinates = [0, 0, 0.3], luminance 70.9 cd/m^2^). Participants were required to press *spacebar* to complete calibration and to begin the trial. Stimuli consisted of six orange circles (radius 0.9°, HSV coordinates = [0.1, 0.5, 0.8], luminance 127.3 cd/m^2^) evenly distributed in a (invisible) circle around a central fixation cross, with a radius of 7.2° (see Fig. 1). After 1,000 ms, all but one of the circles changed colour from orange to red (HSV coordinates = [0.05, 0.5, 0.8], luminance 117.7 cd/m^2^). The target circle was defined as the one circle that maintained the original orange colour. A discrimination target (DT), presented as a forwards or backwards *C,* appeared inside the target circle. Participants were instructed to look directly and as quickly as possible to the target circle. On half of trials an additional red distracter circle would appear, simultaneously with the colour change (see Fig. 1). The distractor circle appeared in-between two existing circles, resulting in six possible distractor locations. The distractor circle was presented at all possible locations equally. Distractor-present and distractor-absent trials were presented in random order. The target array was displayed for 800 ms.

During the experiment, after each trial, participants were asked, via a message displayed on the computer screen (i.e. ‘Was this a good trial?’). Participants responded by pressing a *y* for *yes* or an *n* for no on the keyboard. Before the experiment began, participants were told that the experimenters were interested in filtering out trials in which they made eye-movement errors. They were instructed that a *yes* response meant that during the previous trial their eyes went from the centre of the display directly to the orange circle target, a *no* response meant their eyes did not move *directly* to the target circle. If participants responded *yes*, they were then presented with a second question: to identify if the target *C*, which was presented within the target circle, was facing either forwards or backwards, by typing *f* for forwards or *b* for backwards. When classifying the landing position of saccades, as long the saccade landed within 2.8° of one of the circles, we assigned it to the closest circle.

There were six potential target locations and six potential distracter locations, so with three replications, this gave 108 trials. We included an equal number of trials with no sudden onset distracter to give a total of 216 trials.

### Results

Each participant correctly identified the *C* orientation on at least 95% of the trials. Participants were considerably less accurate in identifying the trials in which they made a ‘good’ eye movement. To analyse this, we categorised the distracter trials based on the total path length of the saccades made by the participant during the trial. Path length was normalised so that one unit represents the distance from the central fixation cross to the centre of the target. We then classed trials in which the total path length was between 1 − *a* and 1 + *a* as ‘good’ (*a* = 0.2 unit). It is important to note that an eye-movement error using this classification method does not necessarily mean that the participant looked at the onset; it only means they did not look directly at the target. The number of ‘capture’ errors versus indirect eye movements that did not land on the onset are shown in Table 1. Saccade latency (i.e. the interval from the colour change to when the eyes started moving) is also shown in Table 1, for completeness. As has been shown in many previous studies, latency is slower when the eyes are correctly directed to the target.

**Table 1:** Number of trials and saccade latency for each category in Experiment 1

	Direct to target	Fixated onset	Other
Number of trials			
Onset	406	658	232
No onset	812	–	484
Mean saccade latency (*SD*)			
Onset	232 (104)	158 (102)	170 (126)
No onset	203 (109)	–	126 (125)

**Fig. 2:**
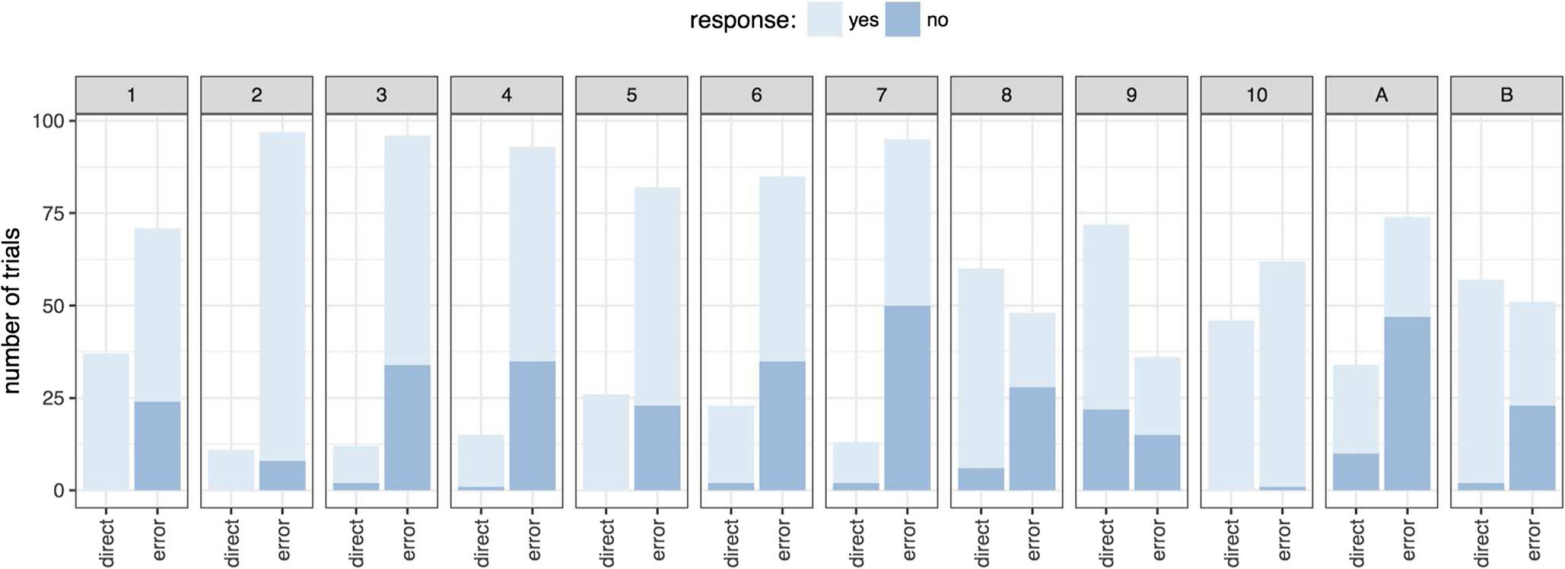
Number of trials in which direct or erroneous eye movements were executed by each participant are shown. Trials which participants identified as good (‘yes’) or bad (‘no’) are presented in light and dark blue, respectively, inside the data bars. Data include only trials on which a sudden onset was presented. (Colour figure online)

Figure 2,^1^ shows the number of distractor-present trials that were classified as good (direct) and bad (error) for each participant, and, within each of these categories, the number of trials to which the participant responded ‘yes’ (it was good) or ‘no’ (it was not). Data from the two authors are presented as *A* and *B.*

**Fig. 3:**
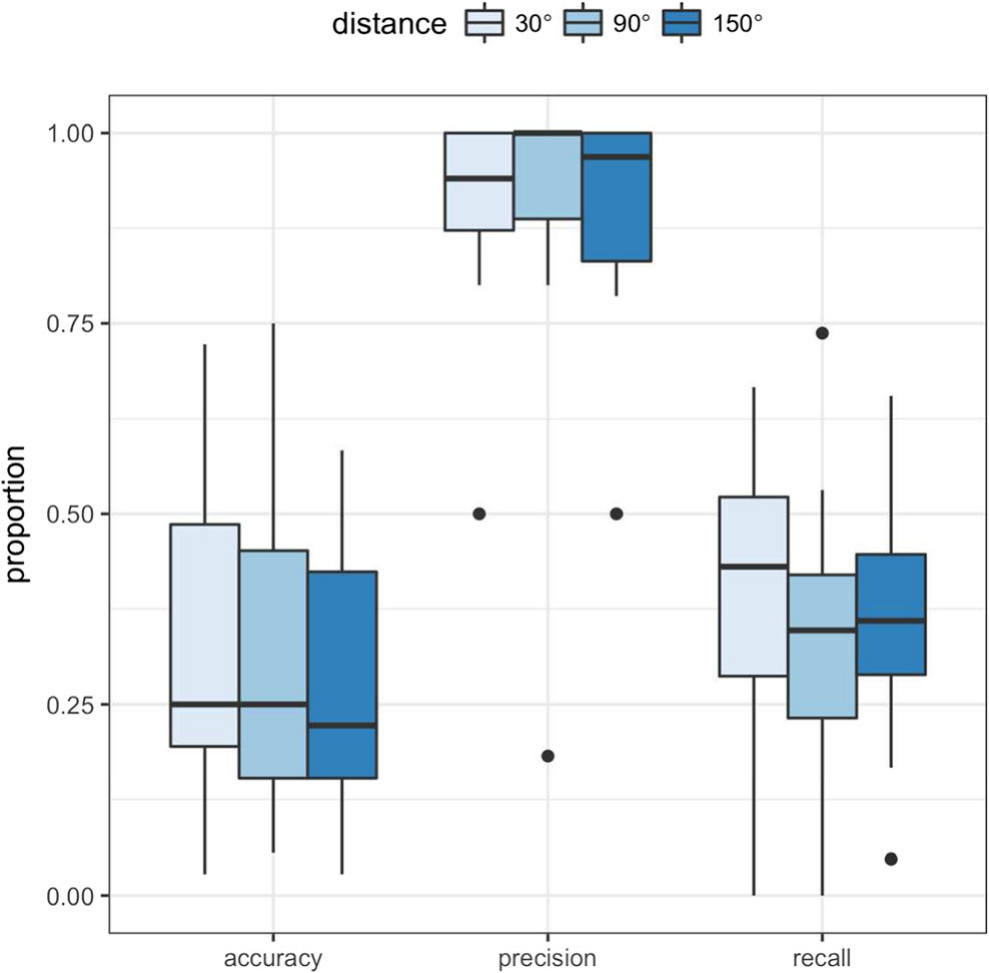
Classification accuracy scores in Experiment 1. Although precision scores are relatively high, recall is low, indicating that many eye-movement errors were not detected. The data are separated by target-distractor distance. The box plots represent the median, first, and third quartiles

It is clear from Fig. 2 that participants varied a great deal in terms of both the accuracy of their eye movements and by how aware they were of eye-movement errors. What stands out in relation to the question at hand, however, is that participants tended to erroneously report a large number of trials with saccade errors as ‘good’. To quantify this tendency across participants, we calculated classification accuracy scores (see Fig. 3). Participants have reasonably good precision scores, that is, around 90% of trials that they reported as not good were indeed trials in which they made a saccadic error. However, median recall is much lower (40%). This tells us that participants are not sensitive to most of the saccadic errors they made during this experiment. Figure 3 shows these scores separately for each target-distractor distance, and it is clear this makes little difference to whether or not participants detect having made an error.

**Fig. 4:**
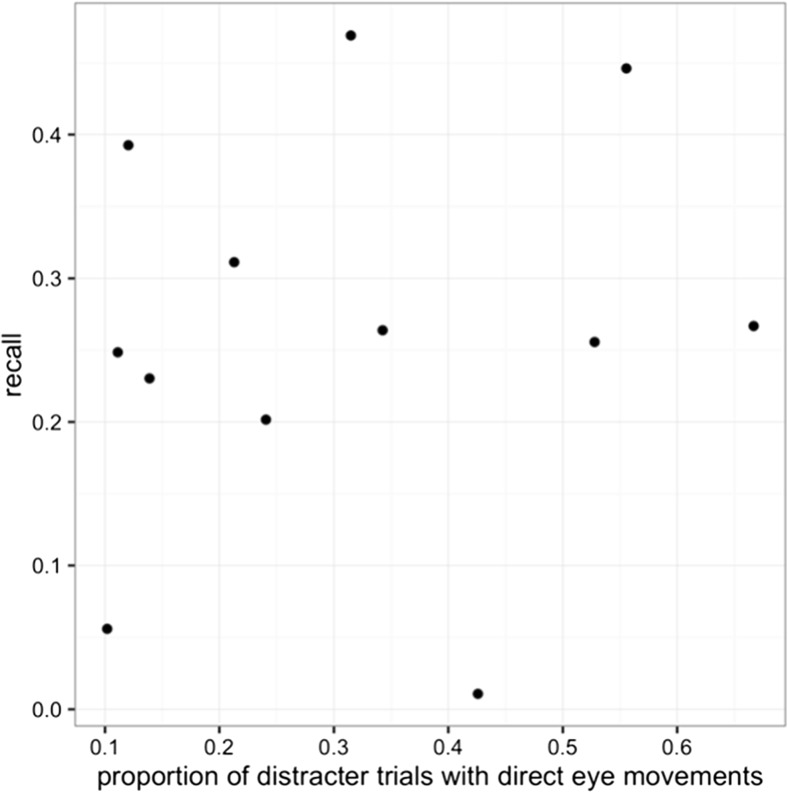
There is no correlation between the tendency to report one’s own errors and eye-movement accuracy

Previous research has suggested that awareness of the onset can either decrease or increase the incidence of capture, depending on the age of the participants (Kramer, Hahn, Irwin, & Theeuwes, 1999). Chisholm and Kingstone (2014) also showed that explicitly telling participants to avoid capture can lower the rate of capture. We therefore checked whether or not awareness of errors was related to the rate of capture in individual participants and found no systematic relationship (see Fig. 4). Being aware of errors does not appear to lead participants to make fewer of them, at least not in our study.

**Fig. 5:**
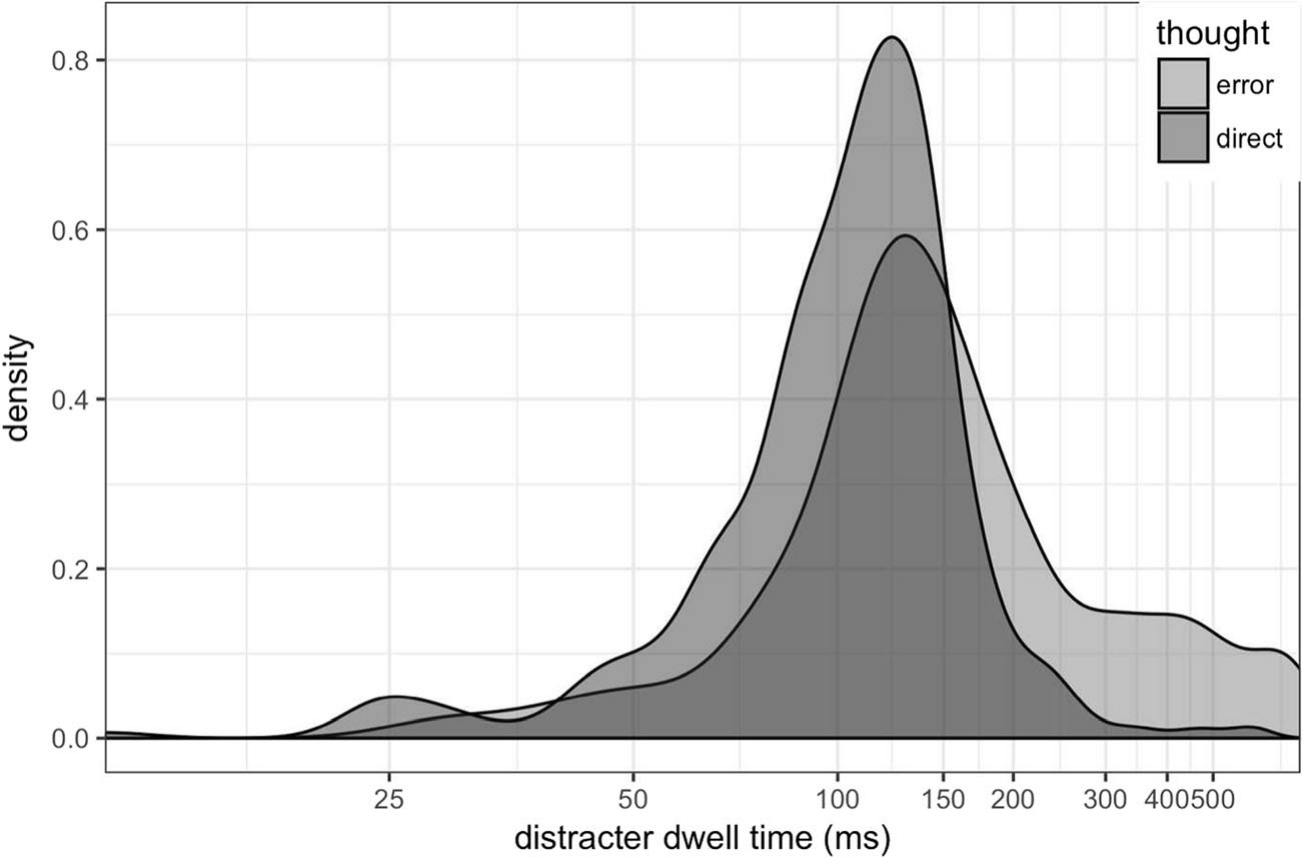
Dwell time on the distracter for trials where the participant reported the error (‘thought error’; light grey) and where they did not (‘thought direct’; dark grey)

Previous research has also found a relationship between dwell time on the erroneously fixated item and reported awareness of the error (Belopolsky & Theeuwes, 2012; Mokler & Fischer, 1999). In Fig. 5, we show the dwell time on the onset distractor on trials in which the error was correctly reported (‘thought error’) and trials in which the participant reported the eye movement was good (‘thought direct’). While there is a great deal of overlap between the distributions, longer dwell times tend to occur on trials in which the error was reported (see Fig. 5). A linear mixed-effects model with a binomial transform showed dwell time, but not saccade latency, is a significant predictor of recall (*p* < .001), with longer dwell times associated with higher recall.

### Discussion

In Experiment 1, eye-movement errors occurred on a large number of trials. The majority of these errors were classified as ‘good’ trials by participants, consistent with the general observation of Theeuwes et al. (1998) that participants are unaware of their errors. Interestingly, even the two authors, who were aware of the motivation for the experiment and the phenomenon of oculomotor capture, were largely unaware of their own eye-movement errors. Awareness of errors and foreknowledge about a tendency for eye-movement errors appears not to reduce the incidence of these errors. Conversely, a tendency to make more or fewer errors was not related to awareness of these eye-movement errors.

## Experiment 2: Attentional capture

In this experiment we explored the contribution of both dwell time and a measure of attention to error awareness. If spatial attention and eye movements are dissociable, as some previous research has suggested (e.g. Hunt & Kingstone, 2003; Stelmach et al., 1997; Van der Stigchel & De Vries, 2015), errors may go unnoticed if the eyes, but not attention, are directed to the onset. To test the role of attention in error awareness, we used a paradigm similar to Experiment 1, but introduced a congruent/incongruent *C* to the target and distractor, as used in Theeuwes and Burger (1998). Participants were required to execute saccades directly to a target singleton and report the orientation of the *C* contained in the target. We expected participants to produce faster reaction times on trials with no onset distractor relative to trials with an onset distractor. On trials with a distractor, we expected participants to also respond slower when the *C* inside the distractor was incongruent with the target *C* relative to when they were congruent. As in the previous study, we should see longer dwell times when participants are aware of their errors than when they are unaware, but with a large degree of overlap. We examined the influence of both dwell time on the distractor and reaction time to the target on eye-movement error awareness, using distractor interference on reaction time as a measure of the extent to which attention was allocated to the distractor. Dwell time and attention may contribute independently to the likelihood of detecting an eye-movement error. Alternatively, their contributions may overlap (e.g. dwell time may increase both error awareness and congruency effects).

The hypotheses and planned analyses for this study were preregistered on the Open Science Framework (https://osf.io/an8gj/). In the preregistered report at this link, we report the results of a pilot experiment on 16 participants which we ran in order to verify that we would be able to observe congruency effects in our paradigm, and to define, test, and refine the analyses we would apply to the new set of data from 36 participants reported below, which we had not yet seen. The Method and Results sections follow the preregistered plan.

### Planned methods

#### Participants

Thirty naïve participants (25 females, *M*_age_ 20.8, range: 19–25 years) took part in the study. We additionally include data from six undergraduate students who were nonnaïve (these students also helped in data collection, and are labelled *a*–*f* in Fig. 7).

#### Stimuli and procedure

**Fig. 6:**
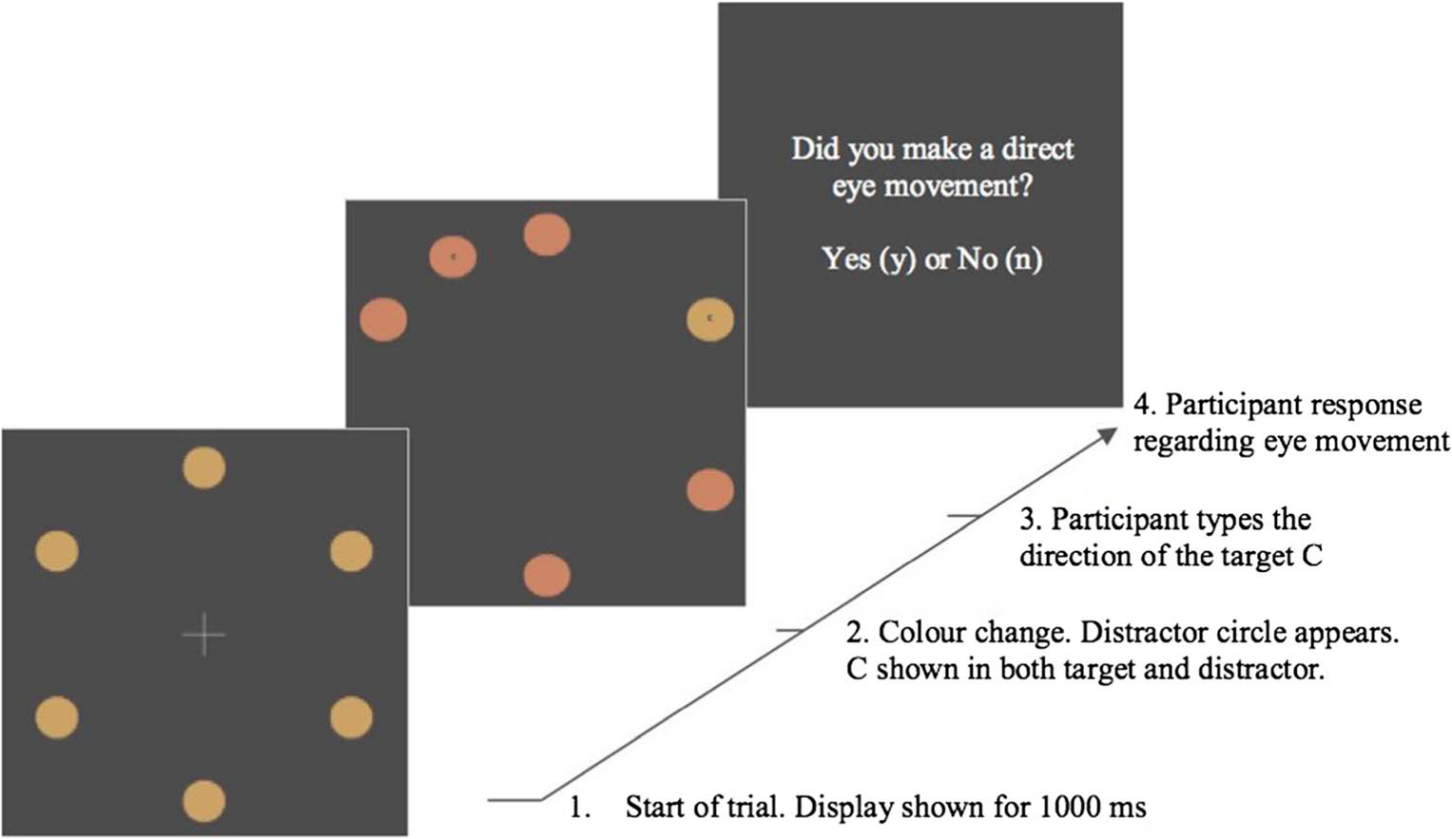
Example of an onset-present trial. Six orange circles are presented in a circular array around a central fixation cross (first panel). After 1,000 ms, five circles change to red, and one remains orange (second panel). As this is an onset-present trial, an additional red circle appears at the same time as the colour change. The distractor circle contains a *C*. Participants must saccade to the orange target and report the direction of the *C* inside the orange target. Participants press the left arrow key for backward or the right arrow key for forward. If they do not respond within 1,500 ms, the screen will display the message ‘too slow’, and the trial ends. Once a response is recorded, participants are then asked, ‘Did you make a direct eye movement?’. Responding ‘yes’ means that the participant believed their eyes went directly to the orange circle on that trial; responding ‘no’ means that they believed their eyes did not go directly to the orange circle on that trial. (Colour figure online)

The general stimuli and procedure were the same as those used in Experiment 1, with two changes (see Fig. 6). First, on distractor trials an additional *C* was presented within the onset distractor. The *C* in the distractor also faced either forwards or backwards (randomly determined). Second, the order of the questions presented at the end of each trial were swapped, with participants identifying the direction of the *C* presented within the target circle first, without being prompted by an on-screen question. They were told to press the key corresponding to the direction of the *C* (left arrow for backward, right arrow for forward) as quickly as possible. If they did not respond within 1,500 ms, the screen displayed the message ‘too slow’, and the trial ended (and was recycled). Following a successful response to the orientation within the deadline, participants were then asked, via a question presented on the screen, whether they had executed a ‘direct eye movement’. In total, the experiment included 577 trials, with half of trials not including a distractor. In distractor trials, the *C* within the distractor was congruent with the discrimination target *C* in the target circle on half of the trials.

#### Planned analysis

Analyses were primarily carried out using mixed-effect models (lme4 1.1–13 library for R 3.4.0), and 95% confidence intervals (CI) on parameter estimates were calculated via parametric bootstrapping using the confint function. All analysis code will be publicly released with the data upon publication (https://osf.io/grbd9/). Following advice from Barr et al. (2013), results are reported from models containing the largest random effects structure that can be support by the data.

#### Modifications from the preregistered plan

This experiment was preregistered on the open science framework (https://osf.io/an8gj/). Relative to our preregistered plan, we made two modifications:

1. We reduced proportion of no-onset trials from 50% to one third. A total of 432 trials were completed by all participants except participant *a*, who completed 648 trials.
2. To clarify what we were asking participants to report, we changed the question about their eye movements from asking whether the eye movement was ‘good’ to asking whether the eye movement was ‘direct’. As can be seen by comparing the results below with the preregistered pilot data, this change did not have any substantial effect on the reporting of eye-movement errors.

### Results

#### Capture rate and error awareness

As in Experiment 1, data presented to illustrate awareness of errors includes all error types (because participants were asked to report whether their eye movement was ‘direct’, any deviations from that are considered errors). The number of trials on which eye movements are directed to the target, the onset and to other locations are shown in Table 2, separately for trials with onsets and no onsets. Saccade latency for each of these conditions is also reported.

**Table 2:** Number of trials and saccade latency for each category in Experiment 2

	Direct to target	Fixated onset	Other
Onset	4144	4158	2210
No onset	4391	–	865
Mean saccade latency (*SD*)			
Onset	226 (126)	147 (158)	189 (197)
No onset	184 (82)	–	165 (177)

**Fig. 7:**
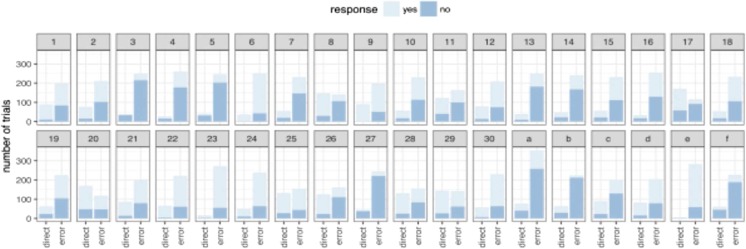
Proportion of trials in which direct or erroneous eye movements were executed by each participant. Trials in which participants identified as indirect (‘no’) or direct (‘yes’) are presented in dark and light, respectively

Figure 7 shows the number of trials with eye-movement errors (using the same criteria as in Experiment 1) and trials with accurate eye movements to the target for each participant. Within each of these categories, the number of trials to which the participant responded ‘yes’ (direct) or ‘no’ (error) is shown.

**Fig. 8:**
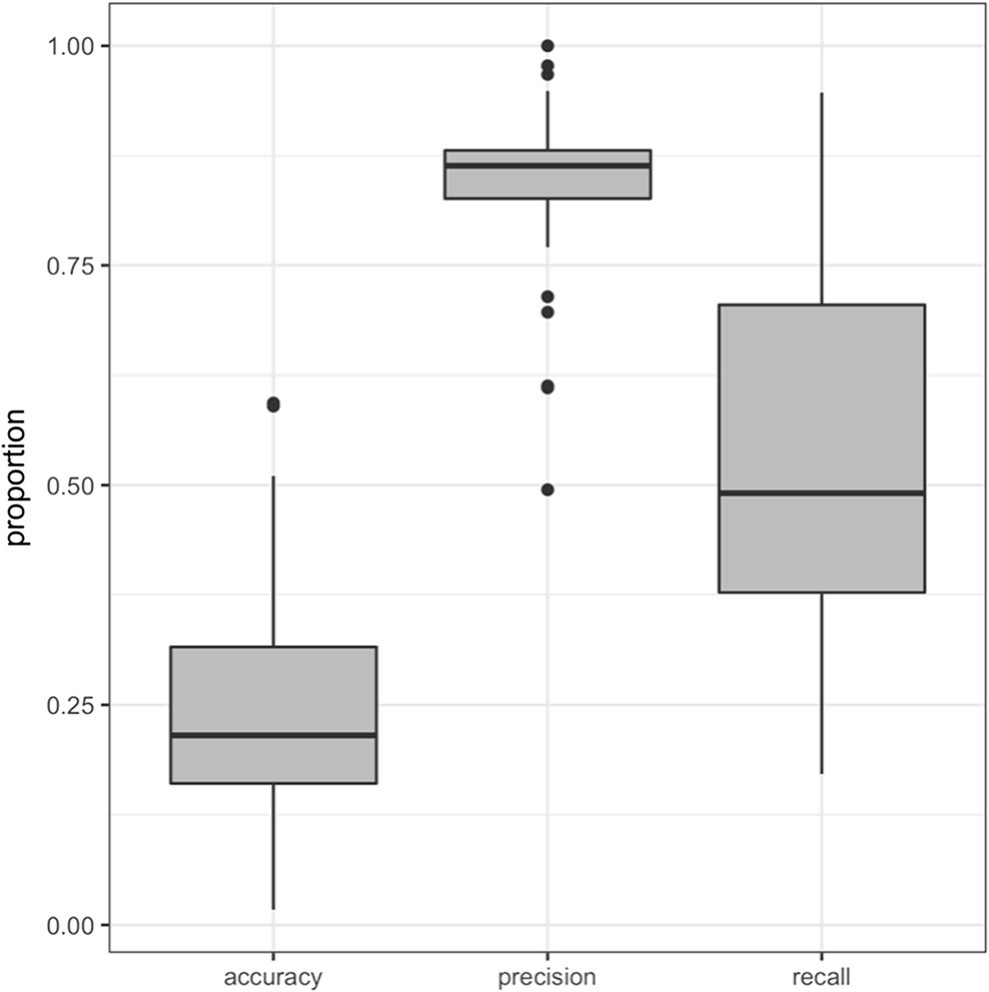
Classification accuracy scores. Although precision scores are relatively high, recall is lower with participants showing a large variation in performance

Classification accuracy scores are presented in Fig. 8. As in Experiment 1, participants make a large number of eye-movement errors. They have reasonably good precision scores, demonstrating that when they think they made an error they are usually correct. But median recall is low, demonstrating that they are not aware of most of their errors. This finding closely mirrors the results of Experiment 1, demonstrating the robustness of this pattern despite several modifications to the procedure (the inclusion of a speeded response to the *C* orientation, the decreased proportion of no-onset trials, the delay in the report of awareness, and the change in wording of the question).

#### Manual responses to discriminate the C orientation

For all the analyses of discrimination reaction time, we removed incorrect trials and trials where the target was not fixated, with 77% of the trials remaining. Accuracy to discriminate the *C* orientation was generally high, with mean accuracies above 89% in all three conditions. Similarly, the average accuracy (over conditions) for each participant was generally high, with all but two achieving an accuracy above 80% (median participant accuracy of 95%, minimum 69%). We verified that the binomial 95% CI around each participant’s accuracy was above 50%.

**Fig. 9:**
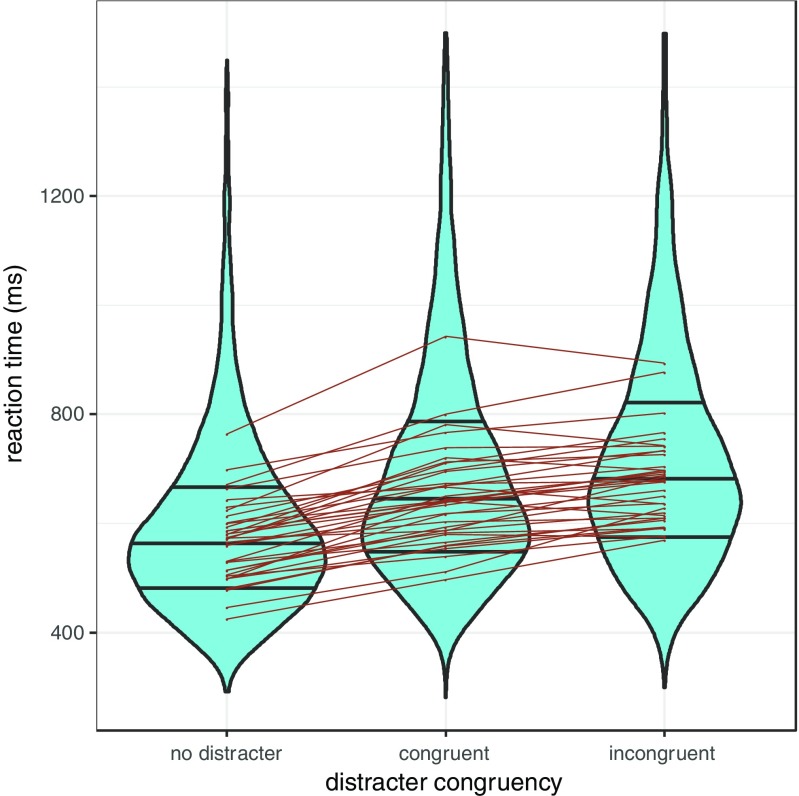
Violin plot depicting the variability of manual reaction time to discriminate the *C* on individual trial data (curve of violins) as well as variability of participant median RTs (points within each violin). For the congruent and incongruent trials, only trials on which participants fixated the distractor onset are included. Participants are slower when there is a distracter onset present, and slower still when that onset contains a *C* that is incongruent with the orientation of the target *C*

In Fig. 9, we present manual reaction time to verify that the sudden onset distractor does slow manual responses, and to further verify that distractor *C* orientation influences responses to the target *C*, with incongruent orientations producing slower responses than congruent orientations.

The presence of the congruency effect was confirmed with a linear mixed-effects model (with maximal random effects structure). As the distribution of response times is skewed, we verify that the 95% CI for the congruency effect size does not contain zero with log-transformed data, but presents the results from the model with untransformed variables for ease of interpretation. We find that reaction times for incongruent trials are 25 ms slower than congruent trials, 95% CI [14 ms, 37 ms]. This is in line with previous findings (e.g. Theeuwes 1991; Theeuwes & Burger, 1998).

#### Dwell time

**Fig. 10:**
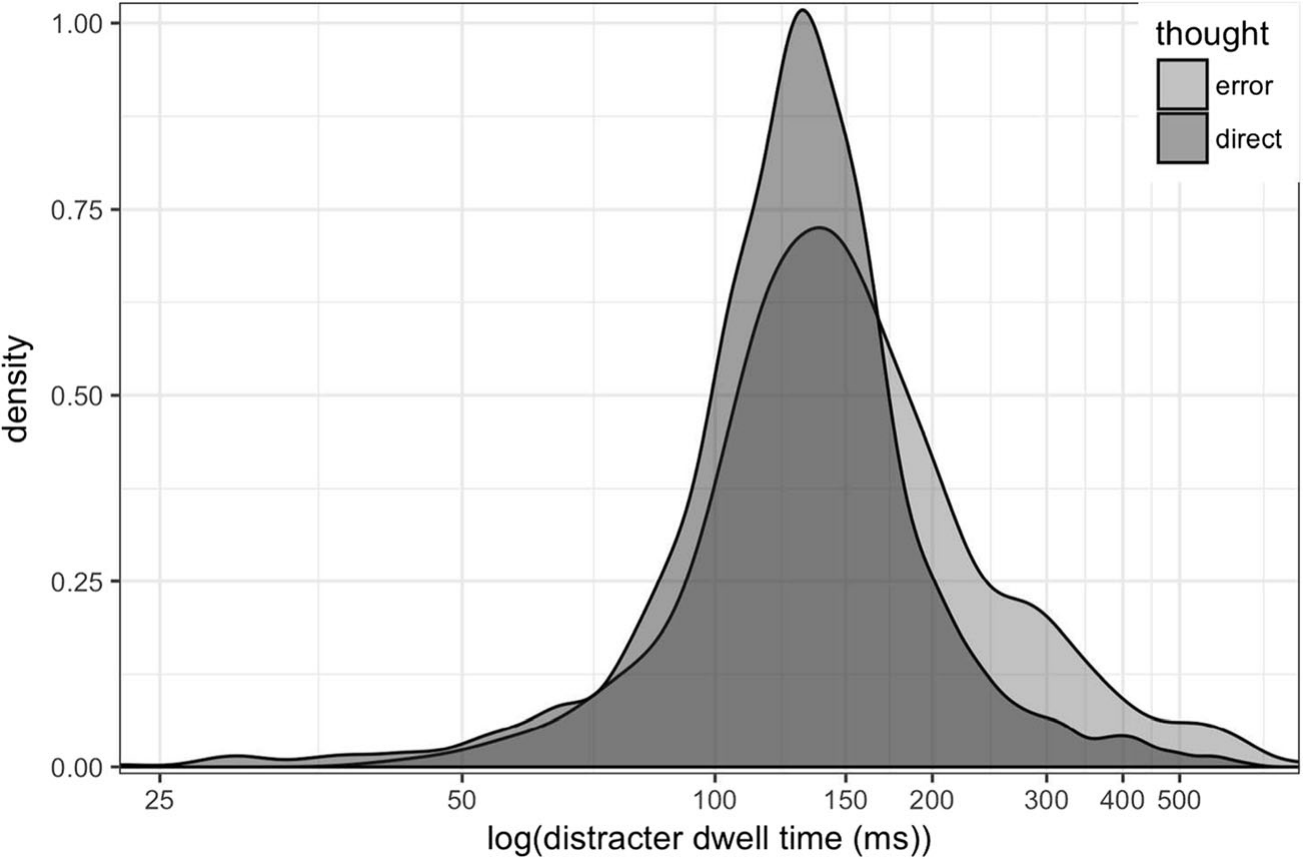
Histogram depicting the dwell time on the distractor onset on trials on which the participant fixated the onset and noticed it (‘thought error’; light grey) versus not noticing it (‘thought direct’; dark grey)

All further analysis is restricted to only those trials with a correct manual response in which the participant fixated both the onset distracter and the target, in that order. This amounted to 3,897 trials (24.7% of all trials). The dwell time on the distractor tended to be slightly longer for those trials on which the errors were reported (see Fig. 10).

#### Predicting error awareness from dwell time and manual RT

We now investigate which factors influence whether participants notice they made an eye-movement error on trials where they fixated first the onset and then the target (24.7% of trials, as above). Specifically, we ran a generalized linear mixed-effects model with a binomial transform to see what influences the likelihood of participants correctly responding that they made an eye-movement error. As predictors, we include the follow factors:

- Whether the trial was congruent or incongruent, coded as a dummy (−1, 1) variable.
- *t*_*d*_: Time spent fixating the distracter (dwell time).
- *t*_a_: Additional response time, defined as manual RT minus dwell time.

We log transform and scale *t*_*d*_ and *t*_*a*_ so that they have zero mean and a standard deviation of one. Only a random intercept was included in the model, as models containing random slopes did not converge.

Congruent and incongruent trials are included in the current model to examine whether errors are unnoticed if the eyes, but not attention, are directed to the onset, suggesting that covert attention and eye movements are dissociable. Both dwell time on the distractor and additional response time are also included based on the results of Experiment 1, which demonstrated that participants display longer dwell times on trials in which they are aware of their errors compared with when they are unaware, but with a large degree of overlap. The main conclusion from the results of this model is that there was no evidence that congruency has an effect on whether participants notice that they incorrectly fixated the target. The other factors and their interaction are significant: *t*_*d*_ beta = .41, (*SE* = 0.047) and *t*_a_ beta = .76 (*SE* = 0.048) with an interaction between these (beta = −.10, *SE* = 0.044). This shows that dwell time and additional response time influence error awareness with these two factors showing an under-additive interaction effect. This under-additive interaction suggests a delay in the overall time to respond to the target (which includes both dwell time and response delay) increase awareness of the error, rather than both of these components contributing to error awareness independently.

#### Are congruency effects still observed when participants are unaware of the error?

Finally, we investigate whether the congruency effect is modulated by awareness, factoring out any effects of dwell time on the distractor. Specifically, we test whether the effect of congruency on *t*_a_ (response time − dwell time)^2^ is modulated by whether participants reported their error or not. As above, this analysis was conducted only on trials where the participant fixated the onset and then the distractor. If awareness of this error is related to attention to the distractor, we should see larger congruency effects on trials where participants are aware of their error. We fit a linear mixed-effects model with congruency and awareness (i.e. whether they reported the error or not) as predictors, with random effects of awareness and random intercepts over observers. The model finds that *t*_*a*_ is 39 ms (95% CI [24 ms, 51 ms]) slower for incongruent trials. Note that this congruency effect of 39 ms is larger than the response-time congruency effect established above (25 ms) in part because this analysis includes only those trials where the eyes landed on the distractor before going to the target. As we would expect given the logistic regression above, *t*_*a*_ is slower in trials in which the observers noticed that they had made an error (by 103 ms, 95% CI [77 ms, 131 ms]). However, there was no evidence that the congruency effect was modulated by awareness, as the effect size was −4 ms, which is close to zero, 95% CI [−26 ms, 16 ms]. This result establishes that the size of the congruency effect is roughly equivalent whether or not participants were aware of having looked at the distractor.

### Discussion

We aimed to address two key questions. First, to what extent are observers able to detect the occurrence of large eye-movement errors (Experiment 1)? While there is a relatively large amount of variability across individuals, we find that the majority of people are unable to report the majority of their eye-movement errors. Nonetheless, when participants do report having made an error, they are usually correct, suggesting they are sensitive to the occurrence of some errors, but not others. Following directly from this conclusion is our second question: What determines whether an eye-movement error is detected or missed (Experiment 2)? We specifically tested the role of two factors: the duration of the fixation on the erroneously fixated object and how much attention was allocated to that object, as measured by the size of the congruency effect. Fixation duration was associated with awareness of the error, but there was no association between congruency effects and eye-movement awareness.

We based our experiment on previous research from Theeuwes (1991) and Theeuwes et al. (1998) and successfully replicated both their attentional capture and oculomotor capture effects, demonstrating that both attention and the eyes were captured by the sudden onset on a substantial proportion of trials. An interesting question that has yet to be clearly addressed is whether or not these two phenomena of attentional and oculomotor capture are one and the same, or if attention and the eyes are both independently attracted to sudden onsets. Hunt, von Mühlenen, et al. (2007) demonstrated that manual responses and saccades are captured by a sudden onset to a similar extent as long as they are matched in terms of response time (i.e. if manual responses are performed under extreme time pressure, to make them as fast as a typical saccadic response). This suggests a central mechanism operates across multiple effectors to drive responses towards the onset. On the other hand, it is not yet clear that this central mechanism is attention. If shifts of attention *cause* capture of the eyes and other response systems, it should not be possible to observe oculomotor capture in the absence of attentional capture. If we had observed congruency effects in Experiment 2 that were reduced or absent on trials where participants were unaware that they made an eye movement to the distractor, then this would have been clear evidence that attentional and oculomotor capture are dissociable phenomena, and by extension, that attention and eye movements are dissociable as well. However, we found that congruency effects are of a similar size, whether or not participants are aware that they looked at the distractor. This result does not rule out the possibility that attention and oculomotor capture are dissociable phenomena, but it is consistent with the generally well-accepted assertion that eye movements and attention tend to shift together, and provides support for the notion that a common priority map drives both attentional and eye-movement selection (e.g. Bisley & Goldberg, 2010).

Theeuwes et al. (1998) reported that participants were unaware that their eyes were captured by the onset, while Belopolsky et al. (2008) found that participants could report about two thirds of their capture errors. In our experiment, participants were able to accurately report about 25% of their eye-movement errors, and this average was relatively consistent across the two experiments, despite several substantial differences between the experiments, including the addition of the speeded manual response and the wording of the question about the error (i.e. whether it was a ‘good’ or a ‘direct’ eye movement). There was a wide variation of results across individuals in our sample, however, which could account for some of the disparity with previous findings. The existence of this individual variation reinforces the importance of obtaining relatively large samples and conveying variability in as much detail as possible when reporting and illustrating results, as we have done here. We can conclude from our results that most of our participants are capable of accurately reporting at least some of their eye-movement errors. This could be taken as evidence that we have direct awareness of our own eye movements, at least some of the time. However, as we argued in our previous study (Clarke et al., 2017), there are many alternative strategies available to participants that can lead to above-chance performance at reporting on their own eye movements. For example, in our previous study, we showed that participants use their memory of the existence of specific objects in a scene to indirectly infer which objects they fixated. Although the distractor objects in the current study were all circles of the same size and colour, the unique onset was a salient signal, and participants could still indirectly infer that they looked at the onset on the trial on which they remember it having occurred. Consistent with this interpretation, having looked at the onset distractor for a longer duration was associated with being able to report the eye-movement error (see also Belopolsky et al., 2008; Mokler & Fisher, 1999). However, the direction of the relationship between fixation duration and awareness remains to be determined: That is, do participants look at the object for a longer period of time because they are consciously aware of it? Or are they aware of the object because they looked at it for a longer period of time? Our fixation durations varied widely (see Figs. 5 and 10), and this is not an exceptional finding; most studies show a similar degree of variation. A recent paper explored the temporal dynamics of saccade execution and could detect no overarching rhythmicity, and concluded a self-paced mechanism constrained by a postsaccade refractory period could best explain variations in fixation durations during periods of free viewing (Amit, Abeles, Bar-Gad, & Yuval-Greenberg, 2017). In their model, a period of inhibition followed each saccade, sampled from a Gaussian distribution, and was followed by a ‘rebound’ period of elevated saccade probability. We have shown that the fixations on the upper end of the distribution of fixation durations are more likely to be reported, but there is still a great deal of overlap between the duration distributions of aware and unaware trials left to explain.

When we isolated only those trials where the eyes went to the onset distractor first and then to the colour singleton target, we observed slower responses to report the orientation of the *C* inside the colour singleton when the *C* inside the onset distractor was incongruent relative to congruent. It is important to note that although the congruency effect was relatively small, we preregistered the experiment on the Open Science Framework, and the planned analysis we conducted was based on a separate set of pilot data from 16 participants (the data from the pilot can be viewed in full in the preregistered document here: https://osf.io/an8gj). This pilot study’s pattern of results was very close to the current results, with small but significant congruency effects. However, awareness did not mediate the magnitude of the congruency effect. While this is a null result, we observed no hint of an effect across both our pilot experiment (*N* = 16) and the current experiment (*N* = 36), suggesting it is unlikely to be due to a lack of power.

We are assuming that the congruency effect is due to attention allocated to the onset distractor because this is how it has been interpreted in previous research (Theeweus & Burger, 1998). We did not measure the effect of letter distractors at locations other than the onset, however. It is possible a diffuse attention to the display as a whole, as opposed to only the target and the onset, could have produced an interference effect when the two *C*s in the display were incongruent. Although the received view is that attention would be required for discriminating the *C* (e.g. Lachter, Forster, & Ruthruff, 2004) there is also some debate around the stage of processing at which interference between two stimuli can occur (e.g. Egner, 2008), including perceptual or response stages. It is therefore possible that a different measure of attention may produce a different pattern of results in terms of its relationship to awareness of the error. Seemingly consistent with this possibility, Belopolsky and Theeuwes (2012) found a slightly larger N1 for onsets when participants were aware of having made the errors. However, like in our study, fixation durations on the onset were also longer when participants were aware of the error, and N1 amplitudes were correlated with fixation duration, making this relationship difficult to interpret. Future research could, for example, measure the N1 on trials equated for dwell time, given the large amount of overlap in these distributions.

Several theories and models have suggested that attention and awareness are dissociable (e.g. Block, 2007, 2011; Cohen & Dennett, 2011; Koch, 2004; Koch & Tsuchiya, 2007; Kentridge & Heywood, 2001; Koivisto, Kainulainen, & Revonsuo, 2009; Lamme, 2003, 2010; Tononi & Koch, 2008). A broad range of experimental paradigms have demonstrated the existence of attention-related effects in the absence of awareness, including visual masking (e.g. Kiefer & Martens, 2010; Naccache, Blandin, & Dehaene, 2002; Shin, Stolte, & Chong, 2009), crowding (e.g. Faivre & Kouider, 2011; Montaser-Kouhsari & Rajimehr, 2005), continuous flash suppression (e.g. Hsieh, Colas, & Kanwisher, 2011; Jiang & He, 2006;) and sub-threshold presentations (e.g. Bauer, Cheadle, Parton, Müller, & Usher, 2009). While the above research makes a clear case that attention can be allocated without awareness, claims of awareness without attention are more controversial (e.g. Koch & Tsuchiya, 2007; Li, VanRullen, Koch, & Perona, 2002; Mack & Rock, 1998). Cohen, Cavanagh, Chun, and Nakayama (2012) argue against such a ‘double-dissociation’, instead proposing that attention is necessary, but not sufficient, for awareness. They suggest that awareness will follow if a requisite amount of attention is allocated to an object. If this account is correct, it is puzzling that variations in reported awareness in our experiment were not associated with variations in our measure of attention. If more attention increases the likelihood that a stimulus or event will be consciously perceived, one would expect larger attentional effects to be associated with reported, as opposed to unreported, events. While we find no evidence that this is the case, both our measure of attention and our self-report method of determining awareness may be influenced in different ways by random trial-by-trial fluctuations in alertness or motor readiness in such a way as to mask their relationship. Our results, in showing that modulations in awareness are not accompanied by changes in measurable allocation of attention, are therefore an intriguing piece of evidence that awareness does not depend on attention, but further research establishing a similar pattern would be a welcome development for better understanding the relationship between awareness and attention.

### Conclusion

Participants looked at a sudden-onset distractor on a large proportion of trials, and they were unaware of the majority of these eye movement errors. Longer fixations on the distractor were associated with a modestly elevated probability that the error would be reported, but there is a great deal of overlap between the distributions of reported and unreported error fixation durations that remains unexplained. Our measure of attention allocation to the distractor showed no difference between reported and unreported errors. The results suggest attention effects do not depend on awareness, and leave open the question of what determines whether an eye movement error will be detected or not.

## Footnotes

1 Note that this figure differs from the version in the preregistered report of this study. The preregistered version included trials with no onset distractor, and in revisiting the results we decided the version with only onset present trials was more comparable to preexisting literature.

2 There was one data point (out of 3,897) with a negative value for *t*_*a*_. This was removed from the analysis. After removing this point, the smallest value for *t*_*a*_ was 128 ms.
